# Human-like tele-health robotics for older adults – A preliminary feasibility trial and vision

**DOI:** 10.1177/20556683221140345

**Published:** 2022-11-14

**Authors:** Nooshin Jafari, Michael Lim, Aida Hassani, Jennifer Cordeiro, Crystal Kam, Kendall Ho

**Affiliations:** Department of Emergency Medicine, 8166 University of British Columbia, Vancouver, BC, Canada

**Keywords:** Virtual care, tele-health, remote medical examination, assistive technologies, robotics, human-like robot, older adults, independent living

## Abstract

**Introduction:**

The global increase of the aging population presents major challenges to healthcare service delivery. Further, the COVID-19 pandemic exposed older adults’ vulnerability to rapid deterioration of health when deprived of access to care due to the need for social distancing. Robotic technology advancements show promise to improve provision of quality care, support independence for patients and augment the capabilities of clinicians to perform tasks remotely.

**Aim:**

This study explored the feasibility and end-user acceptance of using a novel human-like tele-robotic system with touch feedback to conduct a remote medical examination and deliver safe care.

**Method:**

Testing of a remotely controlled robot was conducted with in-person clinician support to gather ECG readings of 11 healthy participants through a digital medical device. Post-study feedback about the system and the remote examinations conducted was obtained from study participants and study clinicians.

**Results:**

The findings demonstrated the system’s capability to support remote examination of participants, and validated the system’s perceived acceptability by clinicians and end-users who all reported feeling safe interacting with the robot and 72% preferred remote robotic exam over in-person examination.

**Conclusion:**

This paper discusses potential implications of robot-assisted telehealth for patients including older adults who are precluded from having in-person medical visits due to geographic distance or mobility, and proposes next steps for advancing robot-assisted telehealth delivery.

## Introduction

The rate of population aging is increasing rapidly in many countries including the United States and Canada,^1^ and is expected to reach a ratio of 1 older adult in 5 persons by 2050.^2,3^ This dramatic shift creates urgency for greater access and more timely care for older adults whose health is susceptible to rapid deterioration if access to quality care is compromised due to geography, frailty and mobility. The coronavirus pandemic (COVID-19) imposed social distancing as a way to limit viral transmission,^4–7^ and telehealth is shown as a viable option to provide essential care services for vulnerable older adults to maintain their wellness, reduce their need for accessing acute care services and their risk of contracting COVID.^8–10^

Robotic technology is suggested to elevate healthcare providers’ capability to perform tasks remotely.^11–15^ It can complement and support remote clinical examination, assessment, diagnosis and decision making,^16–20^ alleviating the burden on frontline healthcare staff. Robot-assisted telehealth can foster independent living and quality care both in hospital and at home settings.^21–24^ Furthermore, integration of human-like features into robots can increase patients’ acceptance and improve attitudes towards human-robot interactions and its intended applications.^25^ Humanoid robots can significantly assist vulnerable patients who suffer from various social, mental and health disorders.^26–28^ While evidence is building on robotic use clinically, more testing to advance robotic technology is necessary to demonstrate its usability in clinical contexts

We conducted a *preliminary clinical validation* study of a human-like general-purpose robot (GPR) in a proof-of-concept robot-assisted telehealth service delivery platform. Through a multidisciplinary collaboration between experienced healthcare professionals, researchers, and technology experts and engineers, we assessed the feasibility of the GPR to use a hand-held digital medical device to collect and monitor an individual’s ECG, which has shown to be a key indicator in the early diagnosis of cardiovascular or health complications including but not limited to COVID-pneumonia^29–33^ and other health disorders^34,35^ caused by severe acute coronavirus 2 (SARS-CoV-2). Literature presents technology solutions, including wearable and wireless systems, proposed for *remote* ECG monitoring.^36–40^ However, there is limited empirical research on the feasibility of such systems that are yet to be validated and integrated into the healthcare system as part of the current routine of telehealth workflows. Lastly, the paper presents feedback from the stakeholders who elucidated the conditions under which the GPR would best be able to complete this telehealth task.

## Methods

We developed the following study protocol and described the technological requirements and actors needed. We collected ECG readings and additional observational data throughout and after the sessions from the study participants and participating clinicians via surveys, interviews and operational notes.

### Research regulatory approvals and COVID-19 adherence

Ethical approval was granted by the University of British Columbia Clinical Research Ethics Board (CREB) (UBC CREB number: H21-00149). Institutional Operational Approval was obtained from Vancouver Coastal Health Research Institute. The study and recruitment protocols were designed in alignment with the current provincial, health authority, and WorkSafeBC health guidelines regarding the COVID-related restrictions. A Safe Research Plan was developed to implement the appropriate safety precautions to protect the study participants, clinicians and researchers against COVID transmission.

Informed consent was obtained from all participants prior to the start of any study activities. Appropriate arrangements were made to provide participant privacy and comfort during the medical exams. Further, female participants were paired with a female clinician and researchers to increase comfort.

### GPR technology description

**Figure 1.**
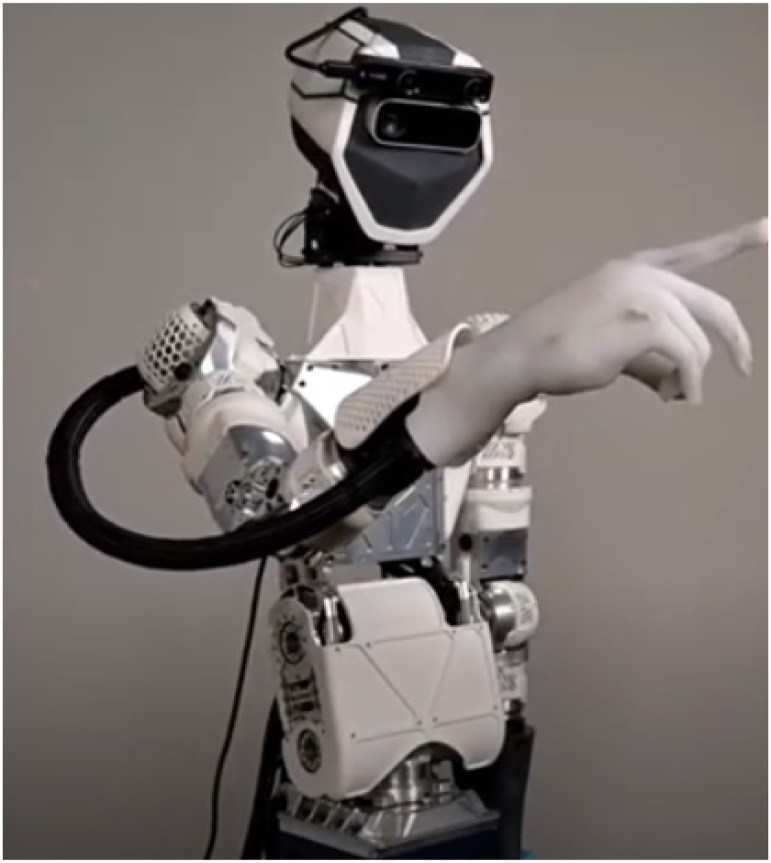
General Purpose Robot (GPR), provided by Sanctuary Cognitive Systems Corporation; Source of image: <https://www.youtube.com/watch?v=nE07hbmH1fg>.

The GPR system, shown below in Figure 1, was provided by Sanctuary Cognitive Systems Corporation. The GPR is configured as a *bilateral teleoperation* consisting of two *haptic* devices/ interfaces - one being operated by the system operator remotely; one in the environment receiving and transferring the interaction forces sensed from the system operator through the teleoperation system.

**Figure 2.**
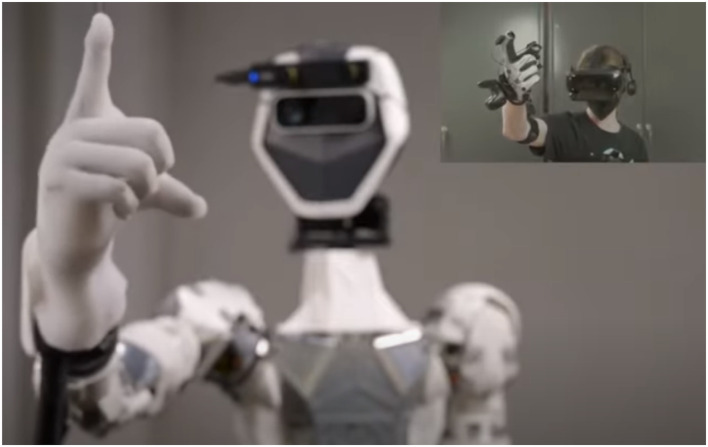
The Pilot, shown in the top right-hand, remotely tele-operates the general purpose robotvia haptic gloves and a head tracker; Source of image: <https://www.sanctuary.ai/product>.

**Figure 3.**
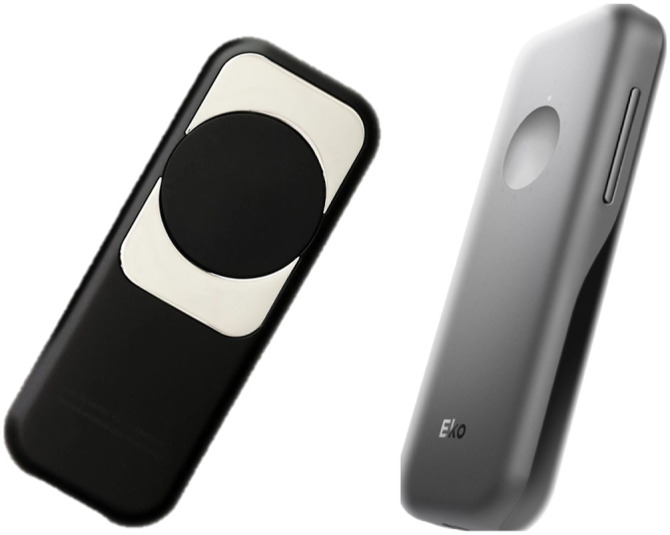
Digital Eko DUO Single-Lead electrocardiogram (ECG) + Stethoscope; Source of image: <https://shop.ekohealth.com/products/duo-ecg-digital-stethoscope>.

To conduct the remote medical exams, the GPR used following components:

1. A commercially available haptic glove as a wearable device to simulate touch sensations of virtual or remote object and to track movements through haptic and motion sensors. As seen in Figure 2, the system operator or the *Pilot* wears the haptic glove.
2. A commercially available consumer-grade Virtual Reality (VR) head tracker worn by the Pilot (shown in Figure 2) to display the 3-D view of the remote examination space where the robotic hand conducts the medical exam on the study volunteer;
3. A commercially available Haptic Glove worn by the Pilot (shown in Figure 2) to simulate touch sensations of virtual or remote objects and track movements through the haptic and motion sensors.
4. A custom-designed and developed human-like robotic haptic hand (i.e. a mechanical hand with built-in haptic sensors) attached to the robotic torso, provided by Sanctuary Cognitive Systems Corporation, remotely controlled by the Pilot via the haptic glove. The robotic hand from torso to fingers was entirely teleoperated by the Pilot.
5. A camera mounted on the GPR to capture the remote examination space and stream the video feed to the Pilot through the head tracker;
6. A commercially available medical-grade and FDA-approved hand-held digital medical device (i.e. Eko DUO, shown in Figure 3) to collect, stream and record ECG signals. The Eko DUO (Eko) is a digital medical device that has shown an ability to detect physiologic abnormalities including ECGs^34,41,42^ and reduce ambient noise in doing so.^43^ The device was held and used by both the study clinicians and the haptic hand in the test sessions.
7. An iPad paired with the Eko device via Bluetooth to stream and display collected ECG recordings in real-time to the clinician.

### Recruitment

Study participants were recruited through a provincial online research platform (https://www.reachbc.ca/). Interested volunteers were eligible to participate if they were 19 years or older, able to attend a one-hour test session in the research lab, and able to sit still in a chair for the duration of exams (i.e. minimum of 3–5 exams). For feasibility testing of the proof-of-concept GPR system and remote medical examination processes during the COVID-19 pandemic, only healthy volunteers with no known existing conditions that could interfere with ECG recordings or their ability to participate safely in an in-person session were engaged. Volunteers were excluded if they were unable to read and understand English, or if they were not able to remove clothing and jewelry from the chest regions during the exam. All participants were required to pass a COVID screening questionnaire prior to and upon their arrival at the test session. Once a study volunteer expressed interest and screened for eligibility, a consent form was sent out to outline the test procedures and expectations for participation. The researcher also reviewed the study protocol, including the robot-assisted medical exam procedure, and consent form with the volunteer in person at the test session.

### Study actors and robot-assisted experimental procedure

**Figure 4.**
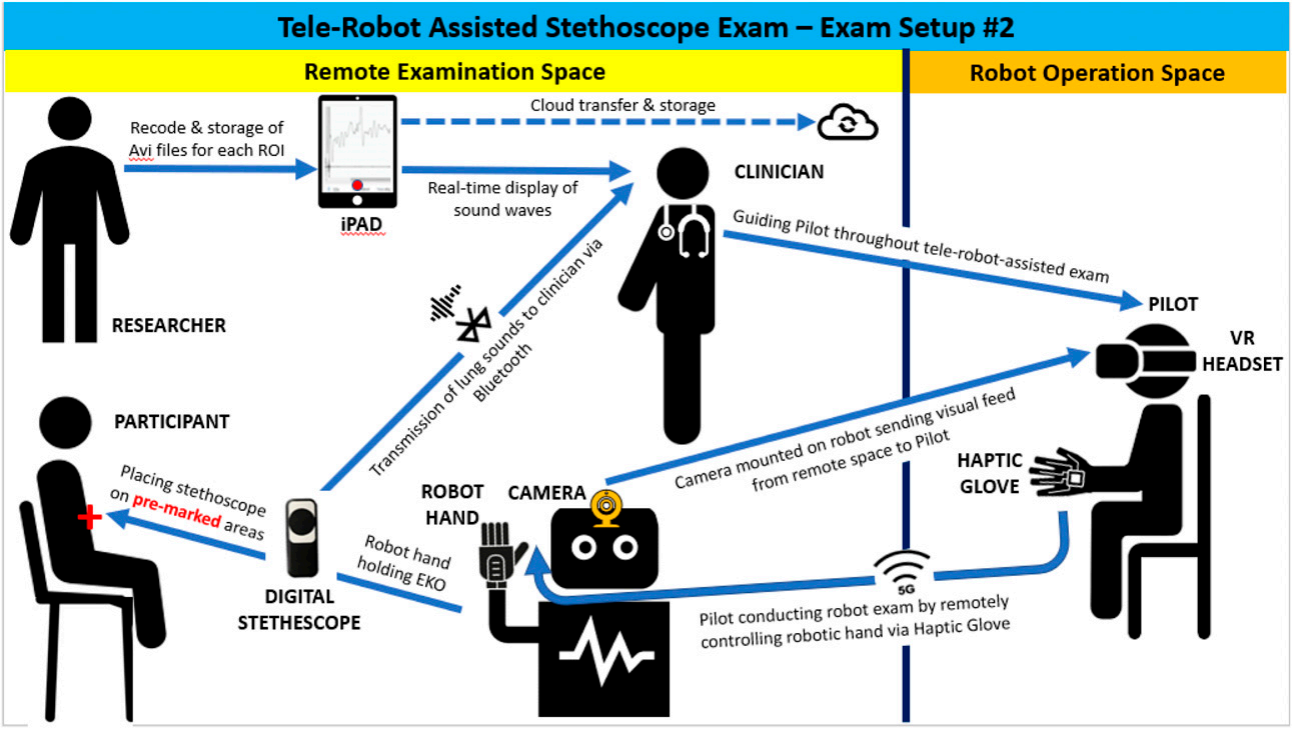
Tele-robot assisted medical examination setup.

The following describes the four types of actors involved in the tele-robot assisted examinations (as illustrated in Figure 4):

1. A healthy human subject in the test session.
2. The study clinician who was situated in the same room as the GPR and participant to observe the examination procedure, ensure participant’s comfort during the study session and verify the quality of ECG recordings. As well, the clinician identified the exam regions of interest (ROI) on the human subject’s torso and provided verbal direction to the Pilot throughout the test session as needed for proper placement, angle and pressure of the Eko device.
3. The Pilot who was located in a space separate from the examination area to remotely conduct the robot-assisted medical exams via the piloting gear and as guided by the clinician through the microphone. As shown in Figure 4, the camera mounted on GPR’s head provided visual feed from the remote examination space to the Pilot.
4. A research study team member who was situated in the same room as the clinician and participant and facilitated the research activities including record and storage of digital data collected by the Eko device, and observed examinations throughout the test sessions.

The clinical study design followed an agile approach in order to adjust to ongoing GPR development and testing cycles. The research protocol was developed based on a single group, non-randomized clinical feasibility trial to scrutinize the capability of GPR system in conducting a medical examination remotely.

Prior to the test sessions, the clinicians were given a demonstration of the GPR system and components, and provided with the Eko medical device to test out. At the start of each session, study participants were asked to remove clothing and jewelry from their chest area. Once ready, they were seated on an adjustable stool chair next to the robot in the remote examination area with the clinician and research study team member. The stool had a back support to allow for safety and stability during the exams.

At the beginning of each session, the study clinician adjusted participants’ chair so as to position them at an optimum distance from the robot and align their upper body with the robot and keep their feet secure on the ground. Once positioned comfortably, the clinician conducted a few hands-on exams using the Eko to identify and mark the desired ROI on the participant’s upper left chest area where a clear ECG signal reading was indicated by the Eko ECG indicator. Once identified, the clinician marked the spot using an erasable surgical marker to provide landmarks throughout the exams to ensure consistency and comparability between the readings.

Once the ROI was identified, the Eko was placed into the GPR hand for the Pilot to begin the tele-operation by placing the Eko device on ROI to conduct ECG recordings. The clinician was able to view the ECG waveforms as streamed in real-time by Eko through the display device (i.e. iPad) held by the researcher. The clinician observed the movement of the robotic hand, and provided verbal cues to the Pilot regarding the placement, angle and pressure of the Eko on the participant’s torso to augment the Pilot’s video feed. The position commands from the haptic glove, worn by the Pilot, were sent to the robotic hand to mirror the same movements. The haptic glove received back forces if/when the Eko was in contact with the participant’s torso. In addition, the VR headset provided visual feed of the examination area to the Pilot. Once the right placement was found, the robotic arm was placed in a “locked” position and stabilized for the “2-min” ECG recording. The locked mode was a safety interlock or “gravity compensation” feature where the system was powered off so the Pilot could effortlessly and steadily hold the robot in position without feeling too much burden on their hand. In case of any fluctuations or changes to the ECG signals during the 2-min examination, the Pilot was able to take over the lock-mode in order to adjust the Eko’s position or applied force. The 2-min timing of the examination was based on the longest duration of ECG recording available via Eko device. Further, the study clinicians verified the adequacy of 2-min recording for making a clinical judgment while alluding the fact that there is no standard length of time for an ECG exam. For proper grip, placement and adjustment of Eko’s pressure against participant’s chest, the Pilot relied on the haptic feedback transmitted via the tele-robot and sensed at their haptic glove. The clinician watched the participants during the readings to ensure there was no pain or discomfort while establishing a balance of optimum pressure and placement of the Eko to get a high-quality ECG waveform.

The feasibility assessment of the system was performed based on the following criteria:

*Exam time*: The duration of “exam time” was based on the start of robot arm movement from the resting position (i.e. at the side of the GPR) to the end of 2-min ECG recording. During the examinations, the research team member tracked the time taken for the GPR to place the Eko and to complete each ECG exam, and compared them against the 2-min baseline. The average time taken for the ECG recordings were calculated to be compared against the 2-min baseline.

*ECG recordings and clinical observations*: Three 2-min ECG readings were collected for each study participant via the GPR system. In order to observe potential variations in the ECG signal, clinicians directed participants to take three deep breaths during the second exam. The other two exams (i.e. exam #1 and 3) were performed under normal breathing conditions. The display device (i.e. iPad), held by the researcher, was paired with Eko device via Bluetooth to display the ECG waveforms in real-time. Eko streamed and recorded the digital data (i.e. ECG readings) in real-time via the cloud-based dashboard (https://ekohealth.com/). The collected data was de-identified and subsequently downloaded into a private secured server network. Field notes were also captured by the study team member to provide additional context to the acquired data and study session outcomes. While the acquired ECG data could be analyzed to help inform the initial assessment of health conditions, this study did not intend to make any diagnosis, clinical interpretations or treatment plans for the study participants. Instead, the accuracy of acquired ECG data was assessed real-time by study clinicians. As well, the use of “ECG signal quality indicator” feature of the Eko application augmented clinician’s observations by providing visual inspection of signal quality.

*Study participants’ feedback*: To help understand the subject’s overall experience, comfort level and acceptance of the system, participants were asked to provide feedback at the end of the test sessions via 5-point scale Likert^44^ and open-ended survey questions. The questions were developed based on an adaptation of the system usability scale (SUS). The feedback was analyzed based on the Likert scale and thematic analysis to identify potential areas of ease or concern.

*Study clinicians’ feedback*: The exploratory post-study open-ended questions were administered with participating clinicians to help unveil clinical perspective on the implications of the GPR technology in healthcare, and inform the ongoing system advancements. The feedback was analyzed based on thematic analysis.

## Results

We recruited 11 healthy adult volunteers (3 females and 8 males) aged 19–49 years old and 3 clinicians in this study. Besides age and gender, participants were asked about their smoking status, height and weight. The average height was 68.7 inches and the average weight was 152.9 pounds. 60% of the participants reported as never smoked and 30% were either former smoker or current smoker. One participant preferred not to answer this question.

The following presents the study outcomes as assessed based on the clinical feasibility criteria.

### Exam time

We collected three 2-minute-ECG recordings for all 11 participants. Due to some technical issues, 3 ECG recordings for one participant did not get properly stored in the Eko cloud database. Thus, a total of 30 recordings were obtained. With respect to the 30 examinations, the average time allocated for each of the tele-robot assisted ECG examinations was 2 min and 54 s.

### ECG recordings and clinical observations

**Figure 5.**
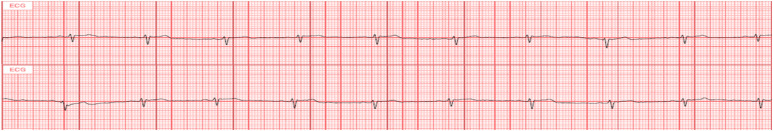
Example of a *normal* ECG signal captured by remote pilot-controlled robot using Eko single-lead ECG device.

**Figure 6.**
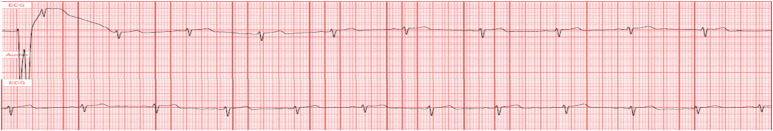
Example of an *interrupted* ECG signal due to motion artifacts caused by sudden temporary shake in remote pilot-controlled robot.

The clinicians verified that the quality of the ECG readings gathered by the GPR using the Eko was generally consistent across all participants, except for 3 unique conditions explained subsequently. The clinicians found the ECG readings easy to interpret and ECG intervals clearly identified. Figure 5 showed an example of *normal* ECG readings where good quality ECG waveforms were acquired with no noticeable irregularities or interruptions. Figure 6 showed an initial artifact due to a sudden movement in the robot hand. This study focused on the quality of the ECG recordings, but not the ability for clinicians to interpret the signals for diagnosis or management. Therefore, clinicians did not comment on any pathologies noted with the ECG recordings.

**Figure 7.**
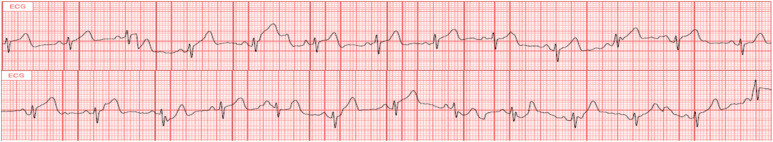
Signal fluctuations caused by lack of consistent contact between body and Eko ECG electrodes.

**Figure 8.**
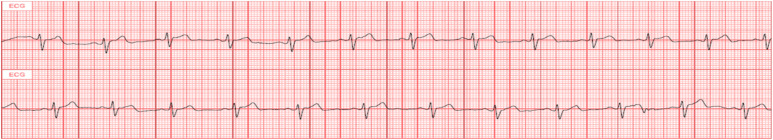
2-min ECG recording of participant with chest hair.

**Figure 9.**
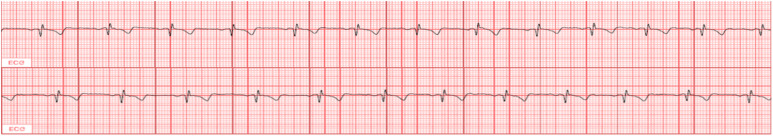
2-min ECG recording of participant with an implant.

We noted three unique physical characteristics of the participants’ chest and torso regions that significantly impacted the ability of the Eko device in data acquisition resulting in variations in quality of ECG waveforms, as assessed in real-time by the Eko ECG signal quality indicator:

1. Participants with low body fat and bony structure: it was particularly challenging to maintain constant pressure while keeping the contact between the ECG electrodes and the body. To find the right ROI, the clinician performed several manual exams and tested a variety of Eko positions (e.g. vertically, horizontally, angled, etc.). During the robot-operated exam, the clinician provided additional guidance to the Pilot on how to grip and firmly press the Eko against the chest to get a stronger signal. The signal quality as shown in Figure 7, however, kept fluctuating between medium-quality and high-quality.
2. Participants with chest hair: body hair caused interference with the ECG signals, resulting in fluctuations and low-quality signals. Similarly, guidance was provided by the clinician to the Pilot to adjust the pressure and find the right ROI. The signal ultimately stayed steady through the 2-min tests (Figure 8).
3. Participants with implants (e.g. breast implant) in left chest area: similar to the situation above, implants blocked signals to the Eko leads, requiring the clinician to perform additional exams in order to determine the right spot and the right amount of pressure required. Extra pressure was applied to get a strong consistent high-quality signal (Figure 9).

### Study participants’ feedback

A feedback survey was administered at the end of the session to help understand participants’ overall experience and collect their feedback on potential limitations and obstacles of conducting a tele-robot assisted medical exams.

**Figure 10.**
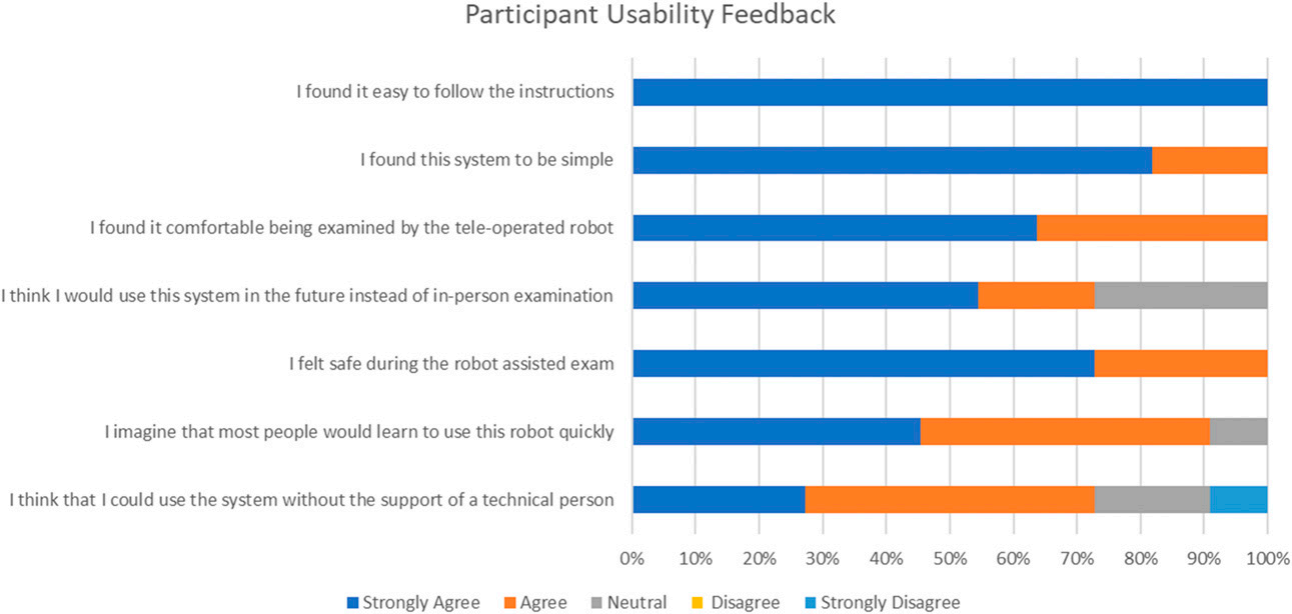
Participant Usability Feedback Results.

On the 5-scale Likert questions (summarized in Figure 10 below), all participants strongly agreed it was easy to follow instructions during the session, and felt comfortable to be examined by the robot. As well, all participants either strongly agreed (73%) or agreed (27%) that they felt safe during the exam. The majority of participants (72.2%) thought they would prefer the remotely conducted medical exam over the in-person examination in the future.

As part of the open-ended questions, participants identified 3 primary factors that contributed to their level of comfort during the test sessions: 1) being familiarized with the GPR Pilot and having the presence of clinician and researchers in the test session room, 2) hearing and being able to communicate with Pilot directly through the mic and camera mounted on the GPR’s head, and 3) having the GPR’s arm locked into position once the ECG signal was detected and stable.

In terms of what reduced their comfort level, the participants indicated occasions of unexpected and/or jittery movement by the GPR, and uncertainty in the level of pressure applied by the GPR during the exam. Overall, participants increased comfort level over the course of the examinations as a result of: 1) an increased familiarity with the test session procedure, 2) developing gradual trust in the GPR during the procedure, 3) finding the GPR personable and mimicking a human being, and 4) easier maneuvering and placement of the Eko by the GPR with practice.

### Study clinicians’ feedback

To help inform the ongoing development of the GPR technology, as well as deliver a safer and more effective system, feedback was collected from the 3 clinicians involved in the study sessions. The clinicians’ perspectives on what worked well were as follows:

- *Humanoid appearance of the GPR*: The silicon hand of the GPR, which resembled human hand/skin, and its ability to move around freely in different angles was seen as a practical asset and the human-like quality of the robot. In addition, the GPR’s ability to move its fingers, grasp the medical device and maintain the right amount of pressure on the chest of the participants increased the applicability of this technology within the clinical context.
- *Precision and endurance*: To collect a high-quality ECG recording, the medical device had to be placed and pressed against the participant’s chest with the right amount of pressure for 2 minutes. The GPR could be directed to successfully perform this task, and once the ROI was found, the arm could be locked for the duration of the reading. One clinician noted that holding the medical device in one place for 2 min could “become tiresome for a real person.”
- *Interactive, real time communication*: The clinician and Pilot were able to communicate via the camera, mic and speaker. This two-way real-time communication was reported to be enormously helpful during the study sessions, increasing comfort and efficiency. Similarly, the Pilot could communicate with the participant as needed (e.g. to adjust distance to the robot or height of the chair). The voice communication kept the interaction dynamic and relevant between the robot and participant.

Clinicians noted the following limitations of the GPR system:

- The current version of the GPR system cannot pick up the medical device on its own and it has to hold the device in a handle which limit its ability to function unassisted.
- The physiological differences in the anatomy of participants could create difficulty in finding the right spot for optimal ECG readings.
- The vibration of the GPR arm could interfere with auditory functions of medical device.

Clinicians also provided additional suggestions on potential clinical settings where this GPR system could be useful:

- *Biohazard containment*: In situations where isolation and critical care is needed (e.g. Ebola virus) or in areas with radiation or other kinds of hazard exposure
- *Rural communities and emergency departments*: In rural and isolated communities, where less resources and healthcare professionals are available, clinicians can remotely visit patients using an automated GPR. This approach could first be tested in urban settings to solve technical and clinical workflow issues, and then deployed in rural contexts.
- *COVID-19 pandemic*: The tele-robotic GPR could lessen person-to-person contact and transmission by performing tasks like: mobile video calls to communicate with and visually examine patients; providing live high-quality imaging; monitoring and reporting patient signs and symptoms.
- *Virtual house calls*: By increasing the mobility of GPR, patients could benefit from such a technology at home with an initial support from a technician. Once the mobile GPR is in a patient’s house, the clinician could remotely visit and/examine the patients. The combined information of a patient’s clinical history and their physical findings can improve diagnostic accuracy and management decisions such as home treatments versus need for hospital visits.
- *Automated clinics*: Use the technology to create accessible and automated clinics where patients can walk in and be remotely examined by a clinician. Ideally, when the patient leaves, the room can clean itself and get it ready for the next patient.
- *Interruption for safety:* Having an “Abort” button in the room that patient can control can increase safety by breaking any motions of the GPR, or even dislocating the arm to prevent unexpected accidents, such as the arm hitting the patients.

## Discussion

This study set out to test the feasibility of GPR system in conducting a medical examination through remote control to safely capture clinical examination data. When robotics can be a safe and trusted tool to conduct remote clinical examinations, it could help bring a clinician’s expertise to patients without exposing either individual to the risk of disease transmission such as COVID. Our study achieved this goal and gathered practical insights from clinicians to shed light on how we can advance this approach for future implementation in healthcare.

From user acceptability perspective, the clinical study protocol was developed with continual feedback from clinicians to ensure maximum comfort and ease-of-use for the study participants. The comfort of participants in being examined by the robot was facilitated through real-time communication with the Pilot and familiarity with the system, to the point that surprisingly the majority would prefer this method to in-person assessment. Future work should be done in similarly interactive fashion so as to seek ongoing end-user’s feedback to improve the GPR’s ease-of-use and perceived acceptability.

Regarding humanoid aspect of the GPR, our study observed the importance of connection between a machine, the participants, and the clinicians. The human-friendly look of the GPR positively impacted the participants’ acceptance. The GPR’s careful non-threatening gestures and movements ‘humanized’ the robot in the eyes of end-users, and the Pilot’s human voice communicating with participants created a GPR-human rapport that enabled participants to feel comfortable being touched and examined by the GPR. Likewise, clinicians commented on the GPR’s humanoid appearance as a plus in providing patient care and facilitating remote communication with patients. While the primary focus of this study was evaluating the system’s feasibility, such discoveries provided compelling evidence on other potentials of the system to be further investigated. A robot with human-friendly gestures, such as head and hand movements, along with human voice integration can make robot-older adult patient interactions less intimidating and more socially engaging while it conducts safe non-invasive examinations and provide care. Such positive effects of humanization of human-robot interactions has been verified in literature.^45^ More research is needed on the social aspects underlying the attitudes and acceptance of GPR, and in reference to the current research in the field, particularly for use cases with older adults in healthcare.

The input from participating clinicians helped identify opportunities to inform future product advancement that address existing healthcare gaps where robotics can improve the delivery of quality care. The key domains were identified as the timely virtual and isolation care, rural and remote care to optimize equity to access resources and to mitigate the viral transmission risks and provide home-based care for patients with limited mobility, and automated clinics with readily-accessible ‘virtual’ clinicians to provide remote care. These potential benefits and opportunities can accelerate transition to more robust telehealth approaches with physical examination incorporated in support of older adults with improved ease of access to optimized care.

The current GPR system requires a skillful Pilot in partnership with the presence of clinicians to accurately perform medical procedures and gain participants’ comfort. With increased system’s maturity, readiness, and autonomy, the ultimate goal is to have clinicians operate the system on their own with no/minimum technical support, thereby providing a more direct and private healthcare provider-patient interaction.

Differences in physiological characteristics of the study participants were found to create hurdles in finding the right ROI for optimal ECG readings. Potential remedies could include applying ECG gel to skin to increase surface contact and improve conductivity, and removing any physical artifacts between the skin and the ECG electrodes (e.g. shaving chest hair). Future studies should consider how robot-assisted remote medical examinations can be successfully performed on participants with a diverse range of physiological characteristics, in particular older adult populations.

In regards to the study limitations, firstly, the clinical research trial was administered when in-person human research was restricted due to COVID-19-related institutional policies to satisfy an ethical imperative to mitigate COVID-19 risks to all humans involved, including the research participants and study team members. Development of a safety plan and modifications to the research protocol were needed to limit in-person and prolonged interactions between individuals so as to ensure appropriate protections for team members and participants. These procedures introduced complexity in the conduct of the study and the speedy of study participant enrollment. As a result, the sample size was small and the trial was restricted to healthy adult participants with no pre-existing medical conditions to mitigate COVID-19-related risks. A future study to include a diversity of individuals in different age groups and with various medical conditions would be desirable. Engaging seniors with health conditions or those with mobility issues (e.g. frail and elderly) will facilitate system and process refinement to tailor this approach to older adults.

This study focused on medical procedures requiring basic movements (e.g. grab, hold and lift) for using the medical tool on easily accessible parts of human body due to the limited mobility of the current GPR arm and hand. Also, the limited maneuverability of the current version of the GPR system restricted its capability to pick up the medical device on its own, therefore requiring someone’s assistance to support placing it in GPR’s hand. Increased agility of a future GPR hand would increase the autonomy of movements. Furthermore, the current GPR system was tethered, immobile. A future GPR system that can be untethered, mobile, and work on wireless or cellular networks would be ideal. With increased system maneuverability, dexterity and improved haptic transparency, the system is expected to be able to carry out full medical examination autonomously.

This initial feasibility study implemented with the GPR technology was essential to grasp the scale of the challenge to get the system ready for fuller-scale clinical usability pilot studies. With increased system’s maturity and readiness, the ultimate vision is to validate the capabilities of a robotic system intervention in autonomously conducting a medical task against a scenario where there is no intervention.

## Conclusion

This paper presented the clinical study design to test tele-robotics technology, and revealed important technical, functional and clinical findings that can contribute to the understanding, advancement, and future deployment of a human-like robotics system in clinical care. The outcomes of this clinical study foster a deeper understanding of the viability of such technologies to address multiple healthcare delivery concerns including timely virtual and isolation care, rural and remote care, remote examinations, contactless triage and remote patient monitoring. The clinical insights collected from the study clinicians and feasibility data inform the development of the system from a laboratory conceptual idea to a pre-clinical system. The integration of humanoid features with robotics was noted to positively impact the human-robot interactions. Further studies as robotics technology advance that include older adults will help to further investigate and validate the technology readiness for formal clinical trial to help uncover whether these discoveries will be promising to support independent living and timely quality care in real world setting. Transitions to robot-assisted telehealth in support of populations who are fragile and in need of optimized access to care requires converging insights and research from professionals in healthcare, science and technology to identify healthcare needs and to design, build and deploy technology solutions to benefit patients and healthcare providers.
